# Supplementary material for: Association between prediagnosis depression and mortality among postmenopausal women with colorectal cancer
Source: PLoS One. 2020 Dec 31;15(12):e0244728. doi: 10.1371/journal.pone.0244728 (PMC7774930; doi:10.1371/journal.pone.0244728)
Supplement: Attachment — Submitted filename: PLOS One paper review_Aug 2020.docx [file pone.0244728.s001.docx]

Thank you for asking me to review this paper entitled: “Effect of depression before colorectal cancer diagnosis on mortality among postmenopausal women” by Liang et al. Using data from the Women’s Health Initiative (WHI) randomized trials and observational study, the authors evaluated the associations between depression prior to colorectal cancer (CRC) and CRC-specific and all-cause mortality.

My main concern with this study is that depression was ascertained only at a single point in time yet depression status changes over time. It would be different if the point in time was meaningful for clinical decision-making (e.g. depression at the time of CRC diagnosis), but in this study the authors are seeking to understand the potential role of pre-diagnosis depression on survival from CRC. Additional concerns are: 1) a lack of information about the elapsed time from ascertainment of depression to the start of follow-up, 2) a lack of information about the source population from which the cohort of women with colorectal cancer arose, and 3) the Discussion section is repetitive.

**Title**

- The use of “effect” in the title implies the findings in this manuscript can be used to make a causal statement. “Association” would be more appropriate for this observational study.

**Abstract:**

- In the methods of the abstract, the authors state that depression was ascertained at “baseline”, however, they do not make clear that “baseline” refers to the definition of baseline in the WHI studies, rather than the start of follow-up in the present study.
- The tense in the first sentence of the conclusion should be past.

**Introduction**

- Lines 72-75: References are needed for each of the specific statements in this sentence, i.e., the specific studies that showed depressive symptoms were associated with an increased risk of CRC and the specific studies that showed that SSRIs were associated with a decreased risk of CRC.
- Lines 78-80: The direction of the association observed by Schofield et al. is not described.
- Lines 89-98: Other than biologic mechanisms, by what means may depression influence CRC survival? More background information about how socioeconomics, health care access, treatment adherence and comorbidities may lead to worse survival in CRC patients would be helpful.
- Lines 91-92: I suggest replacing “organism” with “a person”, if that is accurate.

**Methods:**

- Lines 106-111: More information is needed about the source population for this study, i.e., the WHI randomized trials and observational study. A reference to a prior publication is okay, but more information is needed in the manuscript itself. What were the inclusion and exclusion criteria in the WHI studies? How was postmenopausal defined?
- Line 108-111: What was the rationale for excluding women with CRC identified via death certificate only? Presumably one reason was that the date of diagnosis was unknown, but this is not stated. How many women were excluded for this reason?
- Line 112-114: What was the rationale for excluding women with a prior cancer diagnosis?
- Line 117-119: The statement about generating an indicator variable for persons with a missing family history needs to be clarified. Do the authors mean they included a missing category for the family history variable? If yes, all categories for this variable should be shown in Table 1 or the number with missing values described in a footnote. What was the rationale for creating a missing category (if that was done) for the family history covariate but not for the other covariates?
- Lines 122-127: More information about the ascertainment of the exposure is needed:
  - Over what calendar years was the exposure ascertained in the WHI studies?
  - Was the questionnaire mailed or in-person?
  - When the authors refer to “baseline”, it’s not clear that they are referring to baseline of the WHI studies (as opposed to the start of follow-up in the present study).
- Line 146: How were CRC diagnoses ascertained? What data source was used?
- Line 148-150: The use of the term “censored observations” is not clear. It gives the impression that the record itself was censored rather than the follow-up time being censored.
- Line 152: The lowest age category was all women <70 years? That seems very broad. A description of the age of the source population (see above) may help the reader to better understand this grouping. The lower age limit should be specified.
- Line 153: Regarding the race variable:
- It seems unusual to capitalize “white”.
- Why were all races other than whites grouped together? Can they be shown separately in Table 1 even if they are grouped in the analyses?
- Can the authors provide a rationale for why they considered race a potential confounder in their analyses?
- Lines 151-164: What data sources were used for ascertainment of the covariates? What was the timing for ascertainment of the covariates? Also, some variables need to be defined more specifically (e.g. colonoscopy (ever/never?), physical activity and diet - over what time frame of the woman’s life, etc.)?
- Lines 158-161: What comorbidities were included in the comorbidity index? Here again, a reference to another paper is helpful, but some basic information is needed in the present manuscript.
- Lines 165-167: Duration of antidepressant use was a variable that was included in the models as an adjustment factor (e.g. lines 185-186). How was it possible to adjust for this variable if all unexposed women had the same value (i.e., 0)?
- Lines 167-169: This statement could be more clear. Does “not enrolled” refer to the observational study?

Statistical analysis

- Lines 177-181: A stratified Cox model was used to estimate the HR of all-cause mortality. Should a stratified model also have been used to estimate the HR for CRC specific mortality? Would this increase the comparability of the HRs estimated for each outcome (even though a stratified model may not have been necessary for CRC-specific mortality)?
- Line 179: Why did the authors adjust for age at CRC diagnosis instead of age at baseline in the WHI trial? Age at CRC diagnosis could be in a causal pathway if depression prior to diagnosis predisposes to CRC development at an older or younger age.
- Line 180: Tumor stage is also potentially in the causal pathway between depression prior to diagnosis and mortality if depression prior to diagnosis predisposes to diagnosis with low or high stage CRC.
- Line 185-186: See comment above about adjusting for duration of antidepressant use. How was this possible?
- Line 191-195: Please provide a brief rationale for about each of the sensitivity analyses.
  - Please show the distribution of elapsed time from depression ascertainment to CRC diagnosis. This time is downstream of the depression itself, is there a concern that it could be in a causal pathway?
  - How did the prevalence of depression change in the cohort when the cutpoint was changed (what was the number of women meeting this stricter definition)?
  - It’s not totally clear what “Year 1” is referring to in the last sentence of this paragraph.
- Did this study need to be approved by the institution where the analyses were conducted? I understand that the WHI received IRB approval, but was approval also needed from the institution where these data were analyzed?

**Results:**

- Line 201-204: This sentence could be more clear. Some words seem to be missing (e.g. “more likely to be”…). Can the authors describe the races individually rather than group all races other than whites together?
- Line 214-216: Please describe the distribution of time from ascertainment of depression to CRC diagnosis. It would help to see this in a table. It would also be helpful to see the distribution of age at depression ascertainment.
- Please show the distribution of year of CRC diagnosis in Table 1.

**Discussion**

- Line 224-225: This sentence is not worded clearly.
- Line 228-229: Please check reference #11, it appears to not be correct as it is a commentary that summarizes reference #10.
- Line 232-234: The wording in this sentence is odd because the authors state that their hypothesis is that an association exists, but they tested the hypothesis and their data did not support it.
- As mentioned above “baseline” is used throughout the paper but the timing of the baseline assessment relative to CRC diagnosis is not clear. One could easily confuse “baseline” with the start of follow-up in the present study.
- Line 243-247. If the authors had a concern about residual confounding by age, why did they not try to adjust for age more robustly in a post-hoc sensitivity analysis, e.g., examining the association among younger women only (and adjusting for age within that grouping (e.g. as a continuous variable))?
- Line 230-247: This section, describing the study limitations, appears to be redundant to some extent with the next paragraph (lines 249-264). These two paragraphs could be combined, and the study strengths could be a separate paragraph.
- Women in the intervention arms of the estrogen-only and estrogen plus progestin trials were randomized to receive menopausal hormones. Does use of menopausal hormones influence depression? How might this be a limitation in this study? How does adjustment for treatment arm (“not enrolled, intervention group, or control group”) address this limitation?

**Table 1.**

- Age – it’s not clear that the columns are percentages.
- BMI – appears to be percentages rather than “mean”.
- It would be helpful to see the number of women in each category rather than only percentages.
- Can all race categories be shown?
- Please show the distribution of year of CRC diagnosis.

**Table 2**.

- Using “deaths” rather than “cases” as a column heading would be more clear.
- In the title it’s not clear that baseline refers to the WHI baseline (same comment for Table 3).

**Figure 1**

- First box: what was the earliest year of ascertainment of cases?
