# Supplementary material for: Association between prediagnosis depression and mortality among postmenopausal women with colorectal cancer
Source: PLoS One. 2020 Dec 31;15(12):e0244728. doi: 10.1371/journal.pone.0244728 (PMC7774930; doi:10.1371/journal.pone.0244728)
Supplement: Attachment — Submitted filename: Review-PLOS ONE.docx [file pone.0244728.s002.docx]

**Reviewer's report**

**Title**: Effect of depression before colorectal cancer diagnosis on mortality among postmenopausal women

**Date**: 15/09/2020

Reviewer's report:

This is a paper that might be of interest for those in the related field. I have some comments and suggestions which may improve the quality of the manuscript.

**1. Abstract**

Background:” please remove repeated sentences the aim is so similar to the previous sentence; you should mention why it is important to study post-menopausal women?

Methods: time interval for the assessment of mortality is not clear

Results: it is confusing needs to be revised and please provide some statistical outputs

**2. Introduction**

Why is this topic important in women? Only the availability of the data does not support generating a research question it should be defined scientifically why this research question is the matter among women, any supportive evidence that they are more likely to suffer depression and so on

**3. Results**

Authors refer the reader to the Tables; they need to provide some key results and measures in the text.

**General comments**

Please remove the post-menopausal from the topic since there is no information or results or supportive text about this

The introduction should be revised

Results section are not clear

There is no information about depression symptoms following the colorectal cancer diagnosis; it was important to consider this as a part of the analysis

**Level of interest**: this might be of interest for researchers in this field

**Statistical review**: I have checked and made some suggestions
